# GRAFT: a graph-aware fusion transformer for cancer driver gene prediction

**DOI:** 10.1093/bib/bbaf706

**Published:** 2026-01-11

**Authors:** Sang-Pil Cho, Young-Rae Cho

**Affiliations:** Department of Software, Yonsei University, Mirae Campus, 1 Yonseidae-gil, Wonju-si, Gangwon-do 26493, Republic of Korea; Department of Software, Yonsei University, Mirae Campus, 1 Yonseidae-gil, Wonju-si, Gangwon-do 26493, Republic of Korea; Department of Digital Healthcare, Yonsei University, Mirae Campus, 1 Yonseidae-gil, Wonju-si, Gangwon-do 26493, Republic of Korea

**Keywords:** cancer driver gene prediction, Transformer, graph fusion, PPI networks, multimodal learning

## Abstract

Identifying cancer driver genes is essential for precision oncology, but existing computational methods are often limited by their reliance on single biological networks and their inability to capture long-range molecular dependencies. To address these challenges, we propose GRAFT, a Graph-Aware Fusion Transformer. This framework learns modality-specific features from protein-protein interactions, pathway co-occurrence, and gene semantic similarity using a multi-view graph encoder. These representations are further enriched with two auxiliary feature types: structural encodings derived from network topology and functional embeddings guided by curated gene sets. The integrated features are then processed by a transformer backbone, where a novel edge-attention bias makes the model explicitly sensitive to the underlying graph topologies, enabling the effective modeling of both local and global dependencies. Extensive evaluations demonstrate that GRAFT achieves competitive performance with leading state-of-the-art methods in pan-cancer analysis, while consistently delivering superior predictive accuracy across numerous specific cancer types. More importantly, a functional enrichment analysis of the novel candidate driver genes predicted by our model confirms their strong associations with key cancer-related processes, demonstrating the model’s ability to make biologically plausible discoveries. By delivering a powerful and interpretable framework, our model not only advances the identification of cancer driver genes but also establishes a robust paradigm for multimodal data integration in systems biology. The source codes and datasets are publicly accessible at https://github.com/spcho-dev/GRAFT.

## Introduction

Cancer is a multifaceted genetic disease driven by various somatic alterations, including point mutations, copy number variations, epigenetic modifications and transcriptional deregulation [1, 2]. Among these alterations, only a subset, called cancer driver genes, plays a pivotal role in initiating and sustaining tumorigenesis by conferring selective growth advantages to affected cells [3, 4]. Accurately identifying these driver genes across diverse tumor types is one of the central goals in cancer genomics, as it is fundamental to advancing cancer diagnosis, prevention, and treatment [5].

The increasing availability of large-scale cancer genomics data, particularly from open platforms such as The Cancer Genome Atlas (TCGA) [6], has led to the development of numerous computational approaches aimed at identifying cancer driver genes. These methods can be broadly classified into frequency-based and network-based approaches.

Frequency-based approaches are built on the assumption that cancer driver genes tend to exhibit higher mutation rates than passenger genes [7]. MuSic [8] and MutSigCV [9] estimate background mutation rates to identify significantly mutated genes, while OncodriveCLUST [10] adopts spatial clustering of mutations within protein sequences. Despite their statistical foundation, these approaches often struggle to detect rarely mutated or non-mutated driver genes and are sensitive to the difficulty of accurately modeling background mutation rates, particularly in heterogeneous tumor samples [11, 12].

To overcome these limitations, network-based approaches have emerged as a complementary strategy. Representative methods such as DriverNet [11] and HotNet2 [13] leverage network topology to prioritize genes with a functional impact on downstream expression or within network modules. However, the reliability of these methods is often compromised by noise and incompleteness in the underlying interaction networks [14]. Moreover, many existing approaches rely solely on a single network modality, most commonly a protein–protein interaction (PPI) network, which restricts their capacity to capture the multifaceted relationships among genes.

Recent advancements in deep learning, particularly graph neural networks (GNNs), have provided a powerful framework for integrating multi-omics features with graph-structured biological data. GNN-based methods [15, 16] apply graph convolutional networks (GCNs) to learn gene representations by jointly considering node features and local topologies. However, many supervised deep learning methods require a substantial amount of labeled data, which is often scarce for specific cancer types. To address label scarcity, alternative paradigms have been explored. For example, some approaches used reinforcement learning, such as RL-GenRisk [17], which frames gene identification as a Markov decision process to reduce dependency on known risk genes. Others have leveraged more complex graph structures to capture higher-order functional relationships beyond simple pairwise interactions; for instance, DISHyper [18] employs hypergraph neural networks to integrate and model functional associations from multiple annotated gene sets. While promising, challenges remain in effectively integrating diverse biological data types and modeling complex, long-range molecular interactions using these approaches. Conventional GCNs are inherently constrained by their local message-passing nature, limiting their ability to capture long-range dependencies and the global graph structure. While enhanced diffusion-based frameworks [19] mitigate the limitations of GCNs, they often remain confined to single network structures and lack mechanisms for multimodal data integration. Multi-relational network approaches, such as MODIG [20] and MNGCL [21], have emerged to leverage diverse biological contexts by integrating multiple networks. However, these methods frequently process networks in isolation or fuse features superficially, limiting their capacity to model complex inter-network dependencies or dynamically weigh data significance.

To comprehensively address these challenges, we propose GRAFT, Graph-Aware Fusion Transformer, a novel end-to-end framework for cancer driver gene prediction. GRAFT employs a triple GCN encoder to learn modality-specific features from PPI, gene semantic similarity, and pathway co-occurrence networks. A subsequent attention-based fusion mechanism then generates informative and discriminative gene representations by dynamically weighting the contribution of each network. Furthermore, GRAFT enriches these representations by incorporating both functional context from curated biological pathways and structural information reflecting the topological role of each gene. Finally, a transformer backbone with an edge-attention bias is utilized to effectively model higher-order, long-range dependencies across the entire gene network.

Through extensive evaluation across diverse cancer types, GRAFT demonstrates robust and superior predictive capability compared to state-of-the-art methods in both pan-cancer and cancer-specific prediction scenarios. By consolidating multi-relational biological networks, multi-omics features, and functional priors into a unified end-to-end framework, GRAFT provides a robust and interpretable tool for advancing the discovery of cancer driver genes in precision oncology.

## Materials and methods

### Datasets

In this study, we integrated diverse multi-omics data and biological networks to predict cancer driver genes. The multi-omics features were utilized from TCGA [6], encompassing 16 cancer types as detailed in EMOGI [15] and MTGCN [16]. Following the feature construction protocol established in these studies, three distinct metrics were computed for each gene across all 16 cancer types: (i) a gene mutation rate, calculated from somatic mutation data; (ii) a differential DNA methylation level, representing the average methylation difference between tumor and matched normal samples; and (iii) a differential gene expression rate, derived from $log_{2}$ fold-changes in expression values. These three metrics were systematically calculated for each of the 16 cancer types and concatenated, resulting in a comprehensive 48D feature vector ($16 \text{ cancer types} \times 3 \text{ omics features}$) for every gene. Finally, the entire feature matrix was standardized using *Z*-score normalization to ensure all features were on a comparable scale before being used as input for our model.

Biological networks were obtained from two distinct PPI databases: STRING [22] and ConsensusPathDB (CPDB) [23]. The STRING PPI network was preprocessed by removing edges with interaction scores below 0.85, duplicated edges, and self-loops, resulting in a refined graph with 10 251 nodes and 501 008 edges. The CPDB network was filtered by removing edges with interaction scores below 0.5, producing a final graph comprising 12 979 nodes and 630 322 edges.

We further incorporated additional functional relationships, specifically semantic similarities between genes derived from Gene Ontology (GO) [24] and pathway co-occurrences based on Kyoto Encyclopedia of Genes and Genomes (KEGG) [25] annotations, both obtained from the supplementary materials of MODIG [20]. Each dataset was filtered to contain only the set of genes shared with the selected PPI network. Edges with semantic similarity scores below 0.8 or co-occurrence relationships below 0.6 were excluded. As a result, as shown in Table 1, when combined with STRING PPIs, the semantic similarity network consisted of 8500 nodes and 409 236 edges, whereas the pathway co-occurrence network included 5830 nodes and 213 244 edges. For CPDB PPIs, the corresponding semantic similarity and pathway co-occurrence networks consisted of 10 328 nodes with 728 616 edges, and 6173 nodes with 225 064 edges, respectively.

**Table 1:** Summary of biological networks with functional relationships used for cancer driver gene prediction

Dataset	Functional relationship	Nodes	Edges
STRING	Protein–protein interactions	10 251	501 008
	Semantic similarities	8500	409 236
	Pathway co-occurrences	5830	213 244
CPDB	Protein–protein interactions	12 979	630 322
	Semantic similarities	10 328	728 616
	Pathway co-occurrences	6173	225 064

To incorporate biological context about gene functions, we used annotated gene sets from the Molecular Signatures Database (MSigDB) [26], following the data curation strategy established in DISHyper [18]. MSigDB comprises gene sets categorized into nine collections; among these, we selected C2 and C5 collections. C2 includes curated pathway information from expert sources such as BioCarta [27], Reactome [28], and KEGG [25], while C5 comprises functional annotations based on GO and Human Phenotype Ontology [29]. In total, the gene sets encompass over 20 000 functional relationships across approximately 17 000 genes. To ensure unbiased model evaluation, gene sets containing cancer-related keywords were excluded during training and testing.

For training in cancer driver gene prediction, we collected positive samples from established cancer gene resources, including Network of Cancer Genes (NCG 6.0) [30], COSMIC Cancer Gene Census (CGC) [31], and DigSEE [32]. Negative samples were defined as genes not listed in NCG, CGC, OMIM [33], and KEGG cancer pathways, after recursive removal. As a result, the final pan-cancer dataset comprised 796 positive and 2,187 negative genes. For cancer type-specific prediction, positive samples were extracted from the cancer-type-specific gene lists in NCG 6.0, while an identical set of 2187 negative genes was consistently applied across all cancer types.

**Figure 1:**
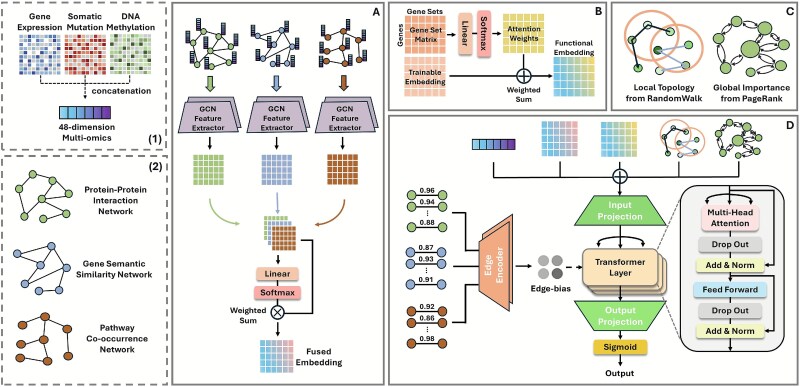
Overview of the GRAFT architecture. (1) Multi-omics features are derived from concatenating gene expression, somatic mutation and DNA methylation data, resulting in a 48D feature vector per gene. (2) Three biological networks—PPI, gene semantic similarity, and pathway co-occurrence—are constructed using STRING/CPDB, GO, and KEGG data, respectively. The GRAFT framework is composed of four main modules. (A) The multi-view graph encoding module extracts node representations from three distinct biological networks (PPI, GO, Pathway) using a separate GCN, which are then integrated via an attention-based fusion mechanism. (B) The functional embedding module guided by gene sets generates functional representations by learning the importance of curated gene sets for each gene. (C) In the graph structural encoding module, local topology features are derived from positional encoding based on a random walk, whereas global importance is determined by PageRank, both computed over the PPI network. (D) The transformer representation learning module concatenates all features and incorporates an edge-attention bias into a graph-aware transformer encoder to capture global dependencies and finally predict cancer driver genes.

### Overview of GRAFT

GRAFT is a comprehensive deep learning framework designed to predict cancer driver genes by integrating diverse biological data sources. The architecture, depicted in Fig. 1, consists of four main stages: multi-view graph encoding, functional embedding, graph structural encoding, and a global graph transformer representation layer. At its core, GRAFT employs multi-view graph encoding (Fig. 1A) to learn rich topological features. It processes three distinct biological networks—PPI, gene semantic similarity, and pathway co-occurrence—using a separate two-layer GCN. The resultant embeddings are then integrated via a learnable attention mechanism to produce a unified, context-rich representation for each gene. To further enrich these representations, GRAFT employs two parallel feature augmentation modules. A functional embedding module guided by gene sets (Fig. 1B) generates functional annotations by learning the importance of curated biological pathways and ontological categories for each gene. Additionally, a graph structural encoding module (Fig. 1C) captures both the global importance of genes, as determined by the PageRank algorithm, and the local neighborhood context of genes through positional encoding based on a random walk within the PPI networks. In the final stage (Fig. 1D), these diverse features—the GCN-fused embedding, the structural and functional augmentations, and the raw multi-omics attributes—are concatenated to form a comprehensive input vector for each gene. This vector is projected and fed into a transformer encoder. To enable the transformer to be explicitly aware of the underlying graph topology, we introduce an edge-attention bias derived from the original biological networks. This bias is incorporated directly into the self-attention mechanism, enhancing the model’s ability to capture meaningful long-range dependencies. The output from the stacked transformer layers is then passed through a final linear layer and a sigmoid function to compute the cancer driver gene prediction score.

### Multi-view graph encoding

To capture complementary biological signals from different functional perspectives, we incorporate a multi-view graph encoding mechanism that integrates three distinct biological networks: PPI, semantic similarity from GO, and pathway co-occurrence. For each network, a separate two-layer GCN is used to extract modality-specific node representations.

Given a graph $ G^{(m)} = (V, E^{(m)}) $ for modality $ m \in \{\text{PPI}, \text{GO}, \text{Pathway} \} $, each node $ i $ has an initial feature vector $ \mathbf{x}_{i} \in \mathbb{R}^{F} $. The two-layer GCN encodes this as:


(1) \begin{align*} \mathbf{h}_{i}^{(m,1)} &= \text{ReLU} \left( \sum_{j \in \mathcal{N}_{i}^{(m)}} \frac{1}{c_{ij}^{(m)}} \mathbf{W}_{1}^{(m)} \mathbf{x}_{j} \right) \end{align*}

(2) \begin{align*} \mathbf{z}_{i}^{(m)} &= \sum_{j \in \mathcal{N}_{i}^{(m)}} \frac{1}{c_{ij}^{(m)}} \mathbf{W}_{2}^{(m)} \mathbf{h}_{j}^{(m,1)} \end{align*}

where $ \mathcal{N}_{i}^{(m)} $ denotes the neighborhood of node $ i $ in graph $ G^{(m)} $ and $ \mathbf{W}_{1}^{(m)}, \mathbf{W}_{2}^{(m)} \in \mathbb{R}^{d \times d} $ are learnable weight matrices. The normalization constant $ c_{ij}^{(m)} $ accounts for the degree-based scaling. As a result, three graph-specific embeddings $ \mathbf{z}_{i}^{\text{(PPI)}}, \mathbf{z}_{i}^{\text{(GO)}}, \mathbf{z}_{i}^{\text{(Pathway)}} \in \mathbb{R}^{d} $ are generated per node.

To integrate information from the three views, we aggregate the modality-specific embeddings into a tensor $\mathbf{Z}_{i} \in \mathbb{R}^{3 \times d} $ and apply a learnable attention mechanism:


(3) \begin{align*} \mathbf{Z}_{i} &= \begin{bmatrix} \mathbf{z}_{i}^{\text{(PPI)}} \\ \mathbf{z}_{i}^{\text{(GO)}} \\ \mathbf{z}_{i}^{\text{(Pathway)}} \end{bmatrix} \end{align*}

(4) \begin{align*} \boldsymbol{\alpha}_{i} &= \text{softmax}(\mathbf{Z}_{i} \mathbf{a}) \in \mathbb{R}^{3} \end{align*}

(5) \begin{align*} \mathbf{z}_{i}^{\text{fusion}} &= \sum_{m=1}^{3} \alpha_{i,m} \cdot \mathbf{z}_{i}^{(m)} \in \mathbb{R}^{d} \end{align*}

where $ \mathbf{a} \in \mathbb{R}^{d \times 1} $ is a learnable attention vector and the softmax promotes interpretability across modalities by highlighting the relative contributions. The fused embedding $ \mathbf{z}_{i}^{\text{fusion}} $ effectively captures the relative importance of each biological view and serves as a comprehensive representation for downstream prediction tasks.

### Feature augmentation by functional embedding and structural encoding

To enrich the node representations with biologically meaningful contextual information, GRAFT incorporates two types of auxilary features: (i) functional embedding guided by gene sets and (ii) positional and centrality encoding. These features are concatenated with node attributes and GNN-derived embeddings to serve as input to the downstream transformer model.

Each gene is functionally annotated using a set of curated biological pathways and ontological categories. To encode the semantic relationship between genes and gene sets, we define a gene set association matrix $ \mathbf{M} \in \mathbb{R}^{N \times S} $, where $ N $ is the number of genes and $ S $ is the number of gene sets. We apply a learnable attention mechanism to compute the importance of each gene set for a given gene:


(6) \begin{align*} & \mathbf{A} = \mathrm{softmax}(\mathbf{M}\mathbf{W}^{\text{attn}}) \end{align*}

(7) \begin{align*} & \mathbf{E}^{\text{gene}} = \mathbf{A} \mathbf{E}^{\text{set}} \end{align*}

Here, $ \mathbf{W}^{\text{attn}} \in \mathbb{R}^{S \times S} $ is a learnable parameter matrix and $ \mathbf{E}^{\text{set}} \in \mathbb{R}^{S \times d} $ is a trainable embedding table for gene sets. The resulting gene-level embedding $ \mathbf{E}^{\text{gene}} \in \mathbb{R}^{N \times d} $ captures the functional relevance of gene sets with respect to each gene. This representation is used to enhance model interpretability and capture functionally informed patterns for prediction.

To encode the structural context of each gene in the PPI network, we employ two complementary strategies:

- **Local topology from random walks**: To capture the local structural context, we first compute a random walk transition matrix by dividing the PPI adjacency matrix entries by their corresponding node degrees. Principal component analysis is then applied to this full $N \times N$ transition matrix to extract the most dominant patterns and reduce its dimensionality. This process yields a variance-scaled positional embedding vector $ \mathbf{e}_{i}^{\text{rw}} \in \mathbb{R}^{d} $ for each gene. This embedding captures the local structural context of the node in a compact form.
- **Global importance from PageRank**: To quantify global influence, we calculate the standard PageRank centrality score $ \mathbf{e}_{i}^{\text{pr}} \in \mathbb{R} $ for each gene, using a damping factor $ \alpha = 0.85 $. This scalar value, inherently normalized between 0 and 1, quantifies the global importance of the node in the network.

To create a unified structural representation, we concatenate the positional embedding and centrality score for each gene:


(8) \begin{align*}& \mathbf{e}_{i}^{\text{struct}} = \left[\mathbf{e}_{i}^{\text{rw}}, \mathbf{e}_{i}^{\text{pr}}\right]\end{align*}

This vector augments each gene’s embedding with both local positional context and global importance, enabling the model to leverage rich structural information from the PPI network.

### Graph representation learning via Transformer

To capture higher-order interactions and global dependencies between genes, we use a transformer-based encoder. For each gene $ i $, we construct a comprehensive input representation by concatenating the raw attributes $ \mathbf{x}_{i} $, the fused multi-view embedding $ \mathbf{z}_{i}^{\text{fusion}} $, the gene set-guided functional embedding $ \mathbf{e}_{i}^{\text{gene}} $ and the structural encoding $ \mathbf{e}_{i}^{\text{struct}} $:


(9) \begin{align*}& \mathbf{h}_{i}^{\text{input}} = \mathbf{x}_{i} \oplus \mathbf{z}_{i}^{\text{fusion}} \oplus \mathbf{e}_{i}^{\text{gene}} \oplus \mathbf{e}_{i}^{\text{struct}}\end{align*}

This concatenated vector $ \mathbf{h}_{i}^{\text{input}} \in \mathbb{R}^{F + 3d + 1} $ is then fused and projected into a shared model space. We employ the following single linear transformation for this step—a strategy deliberately chosen over more complex, gated fusion techniques for its parameter efficiency and robustness against overfitting:


(10) \begin{align*}& \mathbf{h}_{i}^{(0)} = \mathbf{W}^{\text{proj}} \mathbf{h}_{i}^{\text{input}} + \mathbf{b}^{\text{proj}}\end{align*}

where $ \mathbf{W}^{\text{proj}} \in \mathbb{R}^{d \times (F + 3d + 1)} $ and $ \mathbf{h}_{i}^{(0)} \in \mathbb{R}^{d} $.

To guide attention using biologically meaningful relationships, we introduce an edge-attention bias tensor $ \mathbf{B} \in \mathbb{R}^{H \times N \times N} $, where $ H $ is the number of attention heads. This tensor modulates the self-attention weights according to the type and confidence of connections observed in multiple biological networks, thereby allowing the model to emphasize more functionally relevant interactions. Specifically, for each biological network $ G^{(m)} $, edge-level relational information is incorporated directly into the attention mechanism. For each connected gene pair $(i,j)$ in network $m$, the corresponding biological score $score_{ij}^{(m)}$ and pairwise indicators are linearly projected and then passed through a sigmoid activation to produce head-specific attention biases:


(11) \begin{align*}& \mathbf{b}_{ij} = \sum_{m} \sigma\left(\mathbf{W}^{\text{edge}} \, {\mathbf{s}}_{ij}^{(m)} + \mathbf{b}^{\text{edge}}\right)\end{align*}

where ${\mathbf{s}}_{ij}^{(m)}$ denotes a compact pairwise descriptor derived from network $m$, and $\mathbf{W}^{\text{edge}}$ denotes a shared learnable projection. The resulting biologically informed bias tensor $ \mathbf{B} $ is incorporated into the multi-head attention layer, allowing the model to modulate attention weights based on the confidence and type of biological relationships between genes. Such integration enables the transformer encoder to capture high-order dependencies while emphasizing reliable and biologically meaningful interactions.

The projected input features are iteratively refined through a stack of $ L $ transformer layers. At each layer $ \ell $, the node representations are updated as:


(12) \begin{align*}& \mathbf{H}^{(\ell)} = \text{TransformerLayer}^{(\ell)}(\mathbf{H}^{(\ell-1)}, \mathbf{B})\end{align*}

where $ \ell = 1, \dots , L $ and $ \mathbf{H}^{(0)} = [\mathbf{h}_{1}^{(0)}, \dots , \mathbf{h}_{N}^{(0)}] \in \mathbb{R}^{N \times d} $ is the initial projected embedding matrix. Finally, the output vector of the last transformer layer $ \mathbf{h}_{i}^{(L)} \in \mathbb{R}^{d} $ is passed through a linear layer followed by a sigmoid activation.


(13) \begin{align*}& \hat{y}_{i} = \sigma\left(\mathbf{w}^\top \mathbf{h}_{i}^{(L)} + b\right)\end{align*}

where $ \hat{y}_{i} \in [0, 1] $ denotes the predicted probability that gene $ i $ is a cancer driver gene.

### Implementation details

Our model was implemented using Python 3.7, PyTorch 1.9.1, and PyTorch Geometric 2.0.4. The core embedding dimension ($d$) was set to 128 for all feature representations. We employed two distinct sets of hyperparameters for the pan-cancer and cancer-specific experiments. For pan-cancer prediction, the transformer was configured with 3 layers ($L=3$), 4 attention heads ($H=4$), and a dropout rate of 0.1. This model was trained for 30 epochs with a learning rate of $0.001$. For cancer-specific prediction, we used a model with 2 transformer layers ($L=2$), 2 attention heads ($H=2$), and a dropout rate of 0.2, trained for 50 epochs with a learning rate of $0.0001$. Across all settings, the model was trained using the AdamW optimizer with a weight decay of $0.0001$. To address class imbalance, we utilized a Focal Loss with parameters $\alpha =1.0$ and $\gamma =1.5$. We performed a detailed sensitivity analysis to validate our model’s key hyperparameters, including the number of transformer layers, attention heads, embedding dimensions, and the dropout rate. The comprehensive results for the pan-cancer model and a representative cancer-specific model (KIRC) are presented in Supplementary Figs S1 and S2, respectively. These experiments confirm the robustness of our selected configurations

## Results

### Performance on cancer driver gene prediction

We evaluated the performance of the proposed method, GRAFT, using two biological networks from STRING and CPDB. We compared GRAFT with eight representative baseline methods, namely MODIG [20], EMGNN [34], GATOmics [35], ECD-CDGI [19], MNGCL [21], DISHyper [18], DISFusion [36], and TREE [37], which include traditional, homogeneous, and multi-network approaches. To ensure a fair and rigorous comparison, all models were evaluated using the identical multi-omics features and biological networks described in Section Datasets. For the baseline methods, we adopted the optimal hyperparameter settings as reported in their original publications. The evaluation was conducted using 10-fold cross-validation and the average area under the Receiver Operating Characteristic curve (AUROC), area under the Precision-Recall curve (AUPRC), and F1-score with a threshold of 0.5 were used as performance metrics.

Table 2 presents the comparative results of pan-cancer driver gene prediction. GRAFT demonstrated highly competitive, state-of-the-art performance, consistently placing among the top-tier methods across both datasets. On the STRING network, GRAFT achieved the highest AUPRC (0.890) and is tied for the highest AUROC (0.917). On the CPDB network, it delivered robust results by securing the second-best performance in both AUROC and AUPRC. Furthermore, statistical analysis using the Wilcoxon signed-rank test confirmed that the superior performance of GRAFT was statistically significant against the vast majority of baseline methods ($P <.05$), while its standing as a state-of-the-art method was reinforced by its statistically comparable performance to the other top-tier models (see Supplementary Table S1). This consistent top-tier performance underscores the effectiveness and strong generalization capability of our proposed architecture.

**Table 2:** Pan-cancer driver gene prediction results on STRING and CPDB networks. The best results are given in bold and the second-best results are given in underline. The proposed method, GRAFT, demonstrates highly competitive and state-of-the-art performance by consistently placing among the top-tier methods across both networks

Method	STRING			CPDB		
	AUROC	AUPRC	F1-score	AUROC	AUPRC	F1-score
MODIG	0.879 $\pm $ 0.021	0.804 $\pm $ 0.025	0.725 $\pm $ 0.048	0.908 $\pm $ 0.028	0.812 $\pm $ 0.055	0.715 $\pm $ 0.042
EMGNN	0.815 $\pm $ 0.027	0.714 $\pm $ 0.046	0.646 $\pm $ 0.041	0.812 $\pm $ 0.048	0.648 $\pm $ 0.082	0.558 $\pm $ 0.056
GATOmics	0.900 $\pm $ 0.022	0.858 $\pm $ 0.045	0.763 $\pm $ 0.037	0.898 $\pm $ 0.043	0.813 $\pm $ 0.060	0.717 $\pm $ 0.058
ECD-CDGI	0.842 $\pm $ 0.030	0.779 $\pm $ 0.034	0.660 $\pm $ 0.056	0.837 $\pm $ 0.047	0.720 $\pm $ 0.076	0.556 $\pm $ 0.069
MNGCL	0.866 $\pm $ 0.036	0.807 $\pm $ 0.051	0.721 $\pm $ 0.050	0.883 $\pm $ 0.032	0.792 $\pm $ 0.039	0.698 $\pm $ 0.029
DISHyper	**0.917 $\pm $ 0.030**	0.887 $\pm $ 0.045	**0.815 $\pm $ 0.041**	0.923 $\pm $ 0.017	0.865 $\pm $ 0.025	**0.806 $\pm $ 0.027**
DISFusion	0.913 $\pm $ 0.025	0.883 $\pm $ 0.048	0.790 $\pm $ 0.043	**0.930 $\pm $ 0.028**	**0.872 $\pm $ 0.032**	0.784 $\pm $ 0.050
TREE	0.850 $\pm $ 0.016	0.661 $\pm $ 0.025	0.651 $\pm $ 0.019	0.835 $\pm $ 0.019	0.701 $\pm $ 0.021	0.624 $\pm $ 0.017
**GRAFT**	**0.917 $\pm $ 0.025**	**0.890 $\pm $ 0.034**	0.806 $\pm $ 0.034	0.928 $\pm $ 0.019	0.867 $\pm $ 0.025	0.776 $\pm $ 0.034

In addition, we assessed the cancer type-specific driver gene prediction performance of GRAFT on 15 individual cancer types using the STRING network, including kidney renal clear cell carcinoma (KIRC), breast invasive carcinoma (BRCA), prostate adenocarcinoma (PRAD), stomach adenocarcinoma (STAD), head and neck squamous cell carcinoma (HNSC), lung adenocarcinoma (LUAD), thyroid carcinoma (THCA), bladder urothelial carcinoma (BLCA), esophageal carcinoma (ESCA), liver hepatocellular carcinoma (LIHC), uterine corpus endometrial carcinoma (UCEC), colon adenocarcinoma (COAD), lung squamous cell carcinoma (LUSC), cervical squamous cell carcinoma and endocervical adenocarcinoma (CESC), and kidney renal papillary cell carcinoma (KIRP).

As shown in Table 3, GRAFT achieved the highest AUPRC in 14 out of 15 cancer types. In large-sample cases such as BRCA (176 positive genes) and LUAD (152 positive genes), the model demonstrated clear advantages, with AUPRC increases of 4.33% and 5.09% over the second-best method, respectively. In medium-sized cancer types such as STAD (64 positive genes) and UCEC (61 positive genes), GRAFT continued to show strong performance, outperforming the second-best method by 11.08% and 11.43%, respectively. This robust performance also extended to data-scarce scenarios. For instance, in KIRP (22 positive genes), GRAFT achieved a significant gain of 15.09%. Furthermore, in the case of CESC (15 positive genes), the model delivered a highly competitive performance with an AUPRC of 0.721, securing a clear top-tier position that reinforces its stability and high performance even when not achieving the top rank. This result, when viewed alongside its top-ranking performance in the 14 other cancer types, underscores the model’s exceptional stability and consistent high-level accuracy across diverse datasets.

**Table 3:** Cancer type-specific driver gene prediction results on the STRING network. The best results are given in bold and the second-best results are given in underline. The proposed method, GRAFT, outperformed the baseline methods across most of the cancer types

Cancer type	KIRC	BRCA	PRAD	STAD	HNSC	LUAD	THCA	BLCA	ESCA	LIHC	UCEC	COAD	LUSC	CESC	KIRP
Model / AUROC															
MODIG	0.670	0.724	0.600	0.757	0.685	0.843	0.405	0.819	0.712	0.622	0.550	0.600	0.785	0.654	0.720
EMGNN	0.697	0.863	0.838	0.708	0.707	0.734	0.707	0.755	0.724	0.745	0.667	0.734	0.716	0.789	0.722
GATOmics	0.851	0.857	0.893	0.900	0.849	0.884	0.721	0.895	0.796	0.814	0.882	0.859	0.862	0.965	0.934
ECD-CDGI	0.884	0.892	0.947	0.885	0.895	0.877	0.764	0.919	0.839	0.832	0.895	0.851	0.938	0.880	0.956
MNGCL	0.944	0.916	**0.974**	0.913	0.914	0.902	0.838	0.950	0.902	0.872	0.880	0.886	**0.970**	0.927	**0.987**
DISHyper	0.865	0.916	0.947	0.894	0.869	0.853	0.845	0.918	0.881	0.878	0.926	0.875	0.913	0.927	0.924
DISFusion	0.879	0.934	0.915	0.883	0.908	0.892	0.825	0.944	0.873	0.918	0.905	0.881	0.916	0.915	0.809
TREE	0.858	0.752	0.874	0.860	0.861	0.897	0.713	0.948	0.905	0.714	0.900	0.724	0.742	0.630	0.902
GRAFT	**0.973**	**0.955**	0.955	**0.940**	**0.924**	**0.916**	**0.879**	**0.951**	**0.932**	**0.930**	**0.942**	**0.899**	0.961	**0.967**	0.953
Model / AUPRC															
MODIG	0.103	0.273	0.154	0.162	0.196	0.453	0.058	0.338	0.129	0.109	0.127	0.219	0.086	0.038	0.105
EMGNN	0.213	0.653	0.490	0.257	0.379	0.424	0.256	0.341	0.335	0.321	0.286	0.321	0.255	0.351	0.138
GATOmics	0.283	0.597	0.551	0.585	0.554	0.672	0.247	0.685	0.410	0.476	0.612	0.555	0.382	0.364	0.643
ECD-CDGI	0.532	0.719	0.753	0.620	0.677	0.664	0.339	0.751	0.470	0.517	0.657	0.577	0.477	0.611	0.581
MNGCL	0.689	0.773	0.826	0.713	0.739	0.729	0.439	0.833	0.587	0.584	0.698	0.649	0.633	**0.756**	0.749
DISHyper	0.580	0.824	0.714	0.638	0.623	0.633	0.633	0.645	0.553	0.604	0.668	0.594	0.563	0.530	0.617
DISFusion	0.524	0.832	0.729	0.607	0.759	0.746	0.535	0.797	0.589	0.690	0.692	0.652	0.503	0.540	0.549
TREE	0.438	0.502	0.569	0.528	0.673	0.690	0.131	0.809	0.479	0.420	0.726	0.292	0.475	0.022	0.560
GRAFT	**0.754**	**0.868**	**0.883**	**0.797**	**0.795**	**0.784**	**0.668**	**0.839**	**0.660**	**0.735**	**0.809**	**0.713**	**0.708**	0.721	**0.862**
Model / F1-score															
MODIG	0.000	0.000	0.000	0.000	0.000	0.148	0.000	0.000	0.000	0.000	0.000	0.000	0.000	0.000	0.000
EMGNN	0.125	0.560	0.361	0.116	0.345	0.326	0.187	0.276	0.288	0.262	0.164	0.277	0.088	0.142	0.047
GATOmics	0.000	0.382	0.373	0.398	0.257	0.480	0.000	0.506	0.157	0.194	0.449	0.306	0.000	0.000	0.000
ECD-CDGI	0.350	0.634	0.424	0.531	0.549	0.476	0.215	0.604	0.301	0.358	0.513	0.405	0.117	0.500	0.333
MNGCL	0.367	0.646	0.639	0.628	0.631	0.627	0.275	0.675	0.383	0.455	0.607	0.563	0.100	0.200	0.233
DISHyper	0.348	0.736	0.482	0.474	0.507	0.569	0.409	0.479	0.341	0.489	0.436	0.500	0.316	0.145	0.243
DISFusion	0.290	0.711	0.558	0.411	0.659	0.678	0.397	0.669	0.455	0.572	0.572	0.533	0.221	0.200	0.267
TREE	0.410	0.484	0.550	0.462	0.623	0.584	0.048	0.721	0.420	0.473	0.686	0.304	**0.540**	0.000	0.567
GRAFT	**0.653**	**0.782**	**0.845**	**0.728**	**0.785**	**0.724**	**0.573**	**0.772**	**0.552**	**0.642**	**0.731**	**0.590**	0.333	**0.620**	**0.662**

These results demonstrate that the model is capable of robustly integrating multiple biological networks and effectively predicting driver genes regardless of cancer type. Moreover, its ability to maintain high performance across both data-rich and data-scarce scenarios highlights the model’s strong generalization capability and representational power. While several baselines showed good performance for specific cancer types, they often failed to maintain consistency across all settings. In contrast, GRAFT achieved stable, superior AUROC and AUPRC scores across all cancer types, suggesting that it successfully learns biologically meaningful features and retains predictive power even in low-resource conditions.

**Figure 2:**
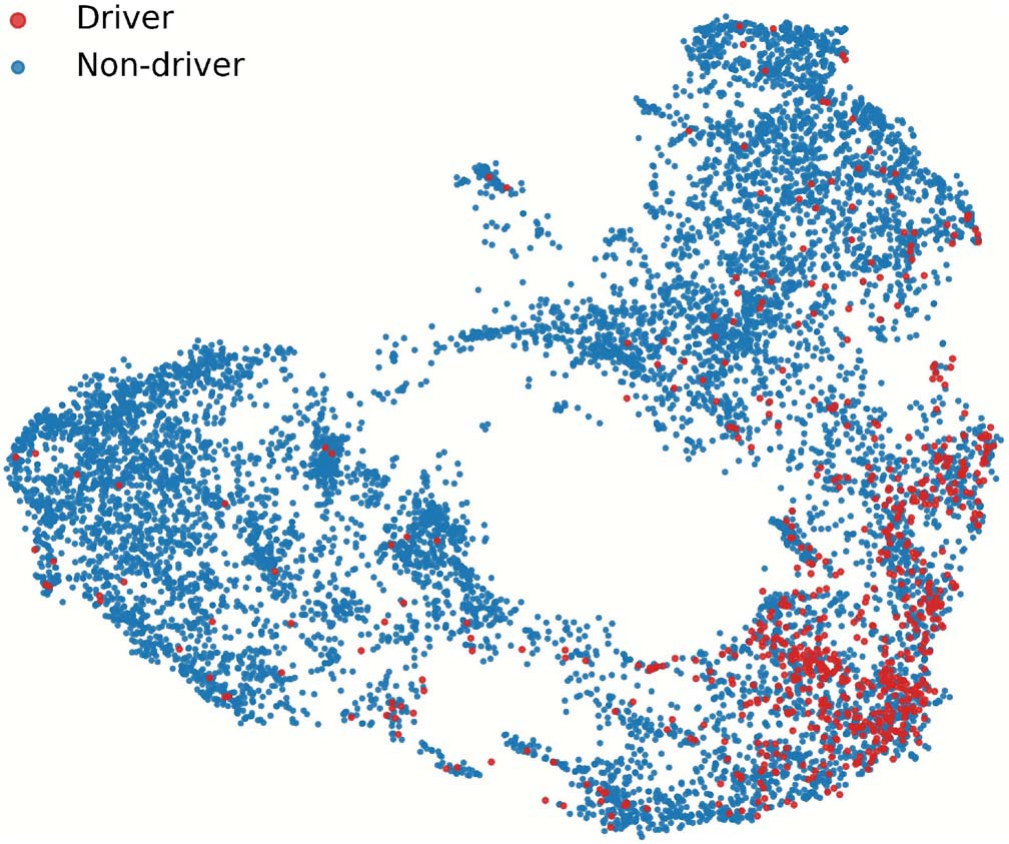
Visualization of driver and non-driver gene embeddings using UMAP. This 2D distribution shows that pan-cancer driver genes tend to form more compact clusters within certain regions of the embedding space.

To further investigate the feature representations learned by GRAFT, we qualitatively visualized the learned embeddings using the Uniform Manifold Approximation and Projection (UMAP) tool. Figure 2 illustrates the 2D distribution of all genes from the pan-cancer dataset, where each point corresponds to a gene and is colored by its ground-truth driver label. As shown, driver genes (in red) tend to form more compact clusters within certain regions of the embedding space, whereas non-driver genes (in blue) are broadly scattered. This suggests that GRAFT is able to project functionally important driver genes into a distinguishable submanifold, indicating its effectiveness in capturing relevant patterns. To quantitatively validate these visual observations, we conducted a comprehensive evaluation using several standard clustering quality metrics. As detailed in Supplementary Table S3, the analysis yielded positive silhouette and high Calinski-Harabasz scores, confirming that the embeddings possess a statistically significant cluster structure despite the biological complexity causing overlap between the two classes.

**Figure 3:**
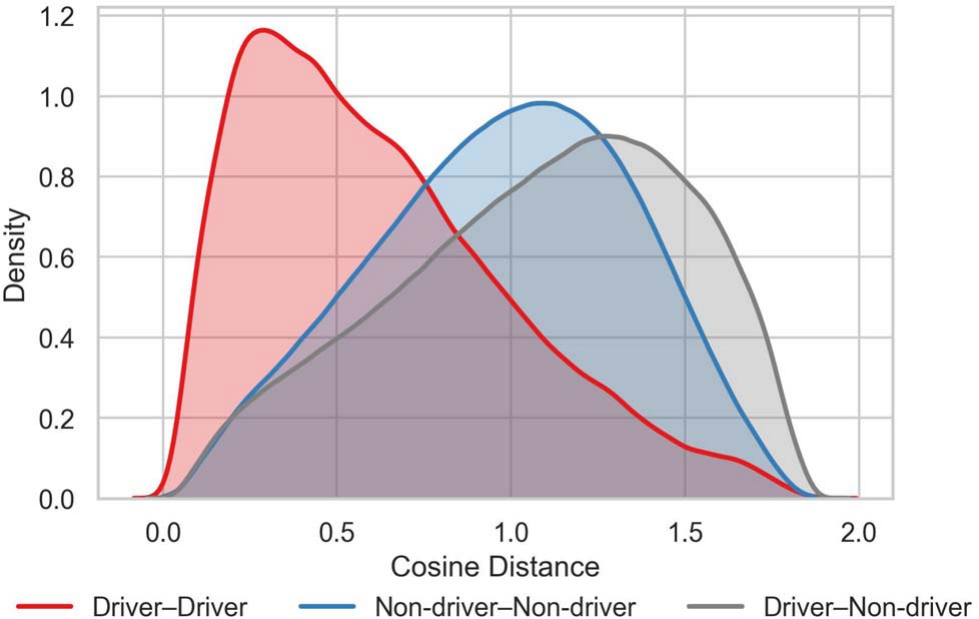
The kernel density estimate of pairwise cosine distances between gene embeddings. The distribution of driver-driver pairs is shifted to the left, peaking at a much lower distance than the other pairs.

Furthermore, we quantified the separability of the embedding space by estimating the kernel density of pairwise cosine distances. The plot in Fig. 3 visualizes the distribution of distances within and between the driver and non-driver groups. Notably, the distribution for driver-driver pairs is clearly shifted to the left, peaking at a much lower distance compared to pairs involving non-driver genes. This visual finding strongly indicates that driver genes are embedded more closely and compactly with one another. This observation is numerically corroborated by the mean pairwise cosine distances reported in Supplementary Table S3, which confirm the trend $Distance(Driver-Driver) < Distance(Non\text{-}driver-Non\text{-}driver) < Distance(Driver-Non\text{-}driver)$. Together, these qualitative and quantitative analyses demonstrate that GRAFT produces a meaningful embedding space that effectively clusters cancer driver genes.

### Ablation study

To assess the contribution of each architectural component in our model, we conducted ablation experiments by systematically removing or modifying specific modules from the full GRAFT architecture. We evaluated performance using AUROC and AUPRC under 10-fold cross-validation on both a pan-cancer setting and a single cancer type, KIRC, known for its limited number of positive samples. The results are summarized in Table 4.

**Table 4:** The ablation results on pan-cancer and KIRC driver gene prediction. The performance on the pan-cancer setting was stable across all variants. However, in the KIRC dataset as a cancer type with limited data, a significant performance decline was observed by individual component removal of GRAFT

Variant	Pan-cancer		KIRC	
	AUROC	AUPRC	AUROC	AUPRC
GRAFT	0.917	0.890	0.973	0.754
GRAFT$_{\scriptsize \text{--PE}}$	0.918	0.894	0.937	0.639
GRAFT$_{\scriptsize \text{--CE}}$	0.918	0.895	0.947	0.685
GRAFT$_{\scriptsize \text{--GSA}}$	0.912	0.885	0.891	0.621
GRAFT$_{\scriptsize \text{--EAA}}$	0.909	0.887	0.954	0.704
GRAFT$_{\scriptsize \text{--NFE}}$	0.918	0.891	0.937	0.695
GRAFT$_{\scriptsize \text{--IF}}$	0.918	0.895	0.939	0.632

Specifically, in GRAFT$_{\scriptsize \text{--PE}}$, the positional encoding module based on a random walk was removed to assess the impact of topological position information. GRAFT$_{\scriptsize \text{--CE}}$ excluded the centrality encoding module to evaluate the role of global importance measures for nodes. In GRAFT$_{\scriptsize \text{--GSA}}$, the gene set attention and embedding module was eliminated, thereby removing the functional context learned from curated gene sets. GRAFT$_{\scriptsize \text{--EAA}}$ disabled the edge-attention bias, which distinguishes edge types during message passing. GRAFT$_{\scriptsize \text{--NFE}}$ excluded the multi-view network feature encoder that produces the fused representation $ \mathbf{z}^{\text{fusion}} $, effectively omitting structural signals from the multiple biological networks. Finally, Graft$_{\scriptsize \text{-IF}}$ removed the initial multi-omics features to evaluate the contribution of the raw input data itself.

According to the ablation results shown in Table 4, the pan-cancer setting exhibited consistently stable performance across all GRAFT variants. AUROC values remained tightly clustered between 0.909 and 0.918 and AUPRC values showed only minor fluctuations, ranging from 0.885 to 0.895. These observations suggest that the full model architecture provides a slight performance gain, but no single component was solely responsible for the overall predictive power in the pan-cancer context. This indicates that GRAFT is robust to individual component removal when trained on abundant and heterogeneous data.

However, in the KIRC dataset, the performance was more sensitive to architectural modifications. The most significant decline was observed in GRAFT$_{\scriptsize \text{--GSA}}$, where AUPRC dropped from 0.754 to 0.621, highlighting the importance of dynamically weighted gene set representations in low-sample scenarios. Similarly, a substantial drop in AUPRC to 0.632 was observed in GRAFT${\scriptsize \text{--IF}}$, where the initial multi-omics features were excluded. This result underscores the critical importance of the raw input data, suggesting it contains fundamental, cancer-specific biological signals that are not fully captured by the derived network or functional embeddings, especially in data-limited scenarios like the specific cancer type. GRAFT$_{\scriptsize \text{--PE}}$ also showed a considerable decrease in AUPRC from 0.754 to 0.639, suggesting that topological positional encoding plays a significant role in preserving structural context, especially under limited data conditions. A noticeable performance drop was also observed in GRAFT$_{\scriptsize \text{--CE}}$, suggesting that centrality signals provide auxiliary value in sparse contexts. GRAFT$_{\scriptsize \text{--EAA}}$ and GRAFT$_{\scriptsize \text{--NFE}}$ resulted in noticeable performance degradation, showing that edge attention and multi-network structural fusion are essential for stable prediction in cancer types with limited data. A Wilcoxon signed-rank test on the 10-fold cross-validation results also confirmed that the full GRAFT model was statistically significantly superior to most ablation variants, as shown in Supplementary Table S2.

### Analysis of gene stratification and interpretability

**Figure 4:**
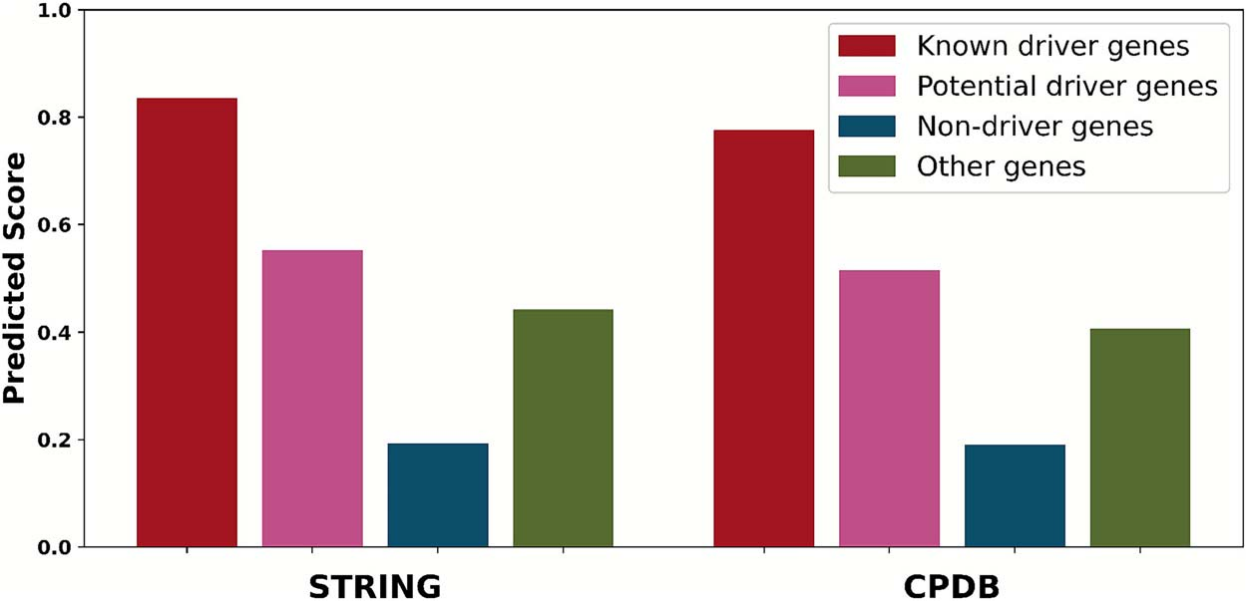
Distribution of predicted scores across four gene categories: known driver genes, potential driver genes, non-driver genes, and other genes.

To assess the capacity to distinguish cancer-relevant genes, prediction scores were analyzed across four gene categories: known driver genes, potential driver genes, non-driver genes, and other genes. Here, potential driver genes represent promising candidates from the NCG database that are not yet established as canonical drivers, and other genes represent the remaining uncharacterized set of genes not included in the three preceding categories. As shown in Fig. 4, known driver genes received significantly the highest scores, followed by potential driver genes. Notably, these potential drivers scored higher than the category of other genes, which in turn received substantially higher scores than non-driver genes. This scoring hierarchy was consistently observed in both the STRING and CPDB networks. This result not only demonstrates the model’s ability to effectively prioritize biologically relevant cancer genes but also highlights its generalization power, as it successfully assigns high scores to potential candidates without direct supervision. The subtle distinction made between non-driver and other uncharacterized genes further suggests its potential as a sensitive tool for novel driver gene discovery.

**Figure 5:**
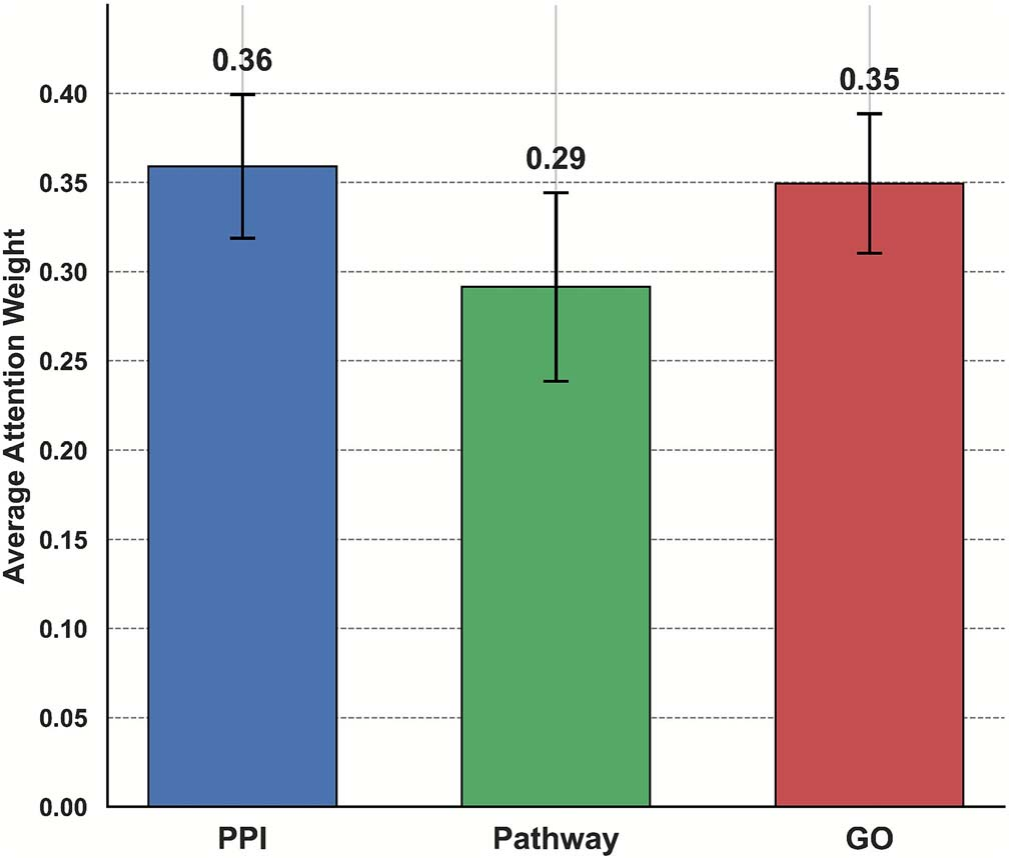
Average attention weights assigned to the three input networks used in GRAFT: PPI, KEGG pathway co-occurrence, and GO semantic similarity.

Next, to understand how the model integrates heterogeneous biological networks, we analyzed the average attention weights assigned to each of the three modalities: PPI, pathway co-occurrence, and semantic similarity from GO. As shown in Fig. 5, the model assigned relatively balanced attention to all three networks, with slightly greater emphasis on PPI and GO sources. These results indicate that the information from PPI and GO contributes more prominently to the feature aggregation process, likely due to their dense and informative connectivity among genes associated with oncogenic functions.

**Figure 6:**
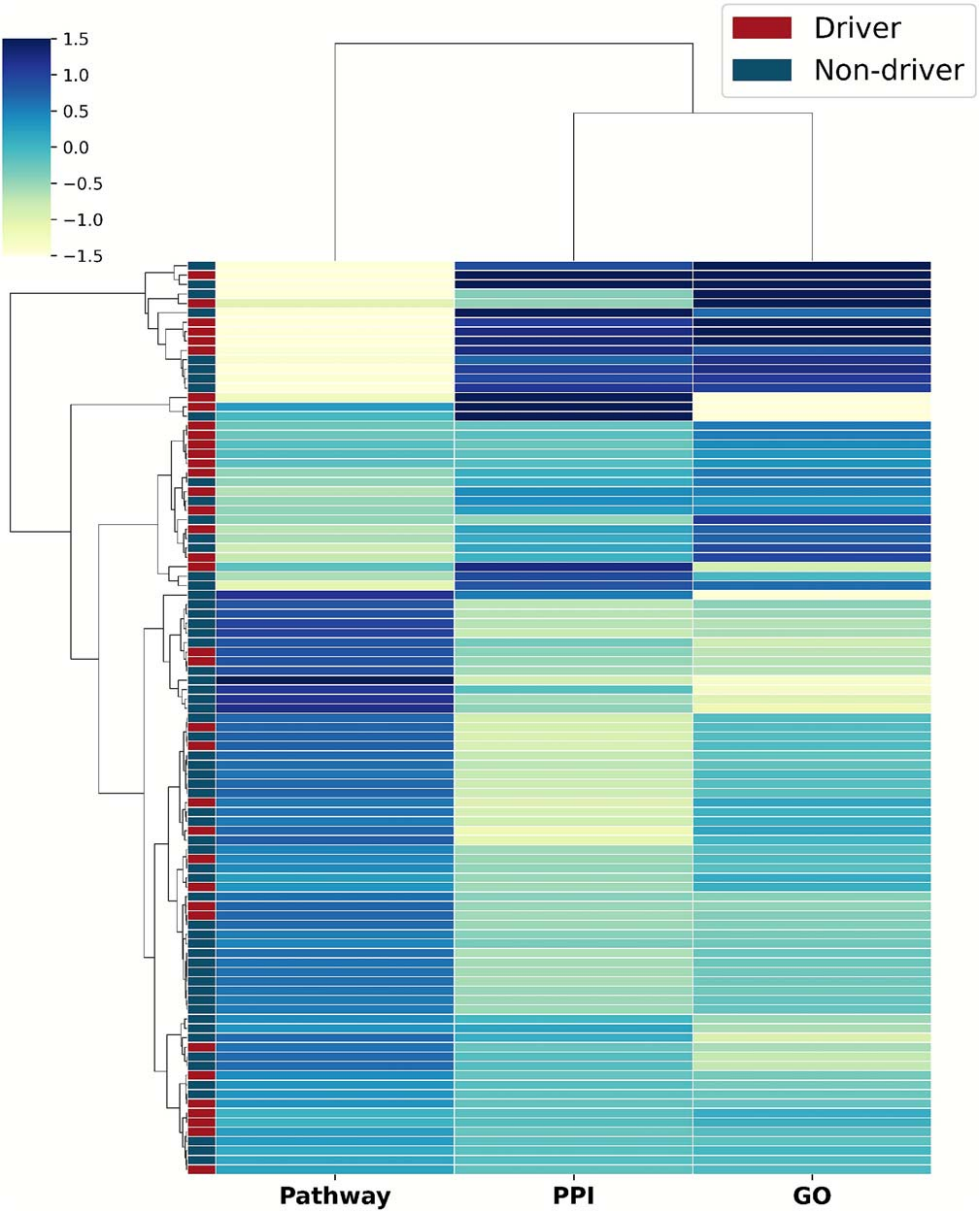
Hierarchical clustering of genes based on their attention weights across the three networks (in columns). Heatmap color indicates relative attention weight; higher intensity corresponds to higher weights. The sidebar visualizes the clustering status of drivers versus non-drivers. Driver genes are distributed across multiple clusters, suggesting diverse archetypes rely on different network contexts for identification.

Lastly, we performed a clustering analysis on the attention distribution profiles of test genes to investigate how the model uses different network modalities. The resulting heatmap in Fig. 6 reveals that genes form distinct clusters based on their attention patterns, such as being PPI-centric or pathway-centric. This indicates that the model leverages network-specific signals in a gene-dependent manner. When mapping known driver and non-driver labels onto this structure, we observed that driver genes do not segregate into a single, monolithic cluster. Instead, they exhibit enrichment within several distinct attention-pattern groups. This finding suggests that there are multiple archetypes of cancer driver genes, each characterized by the type of network context that is most informative for its prediction. For instance, some drivers may be identified primarily through their central role in the PPI network, while others are defined by their involvement in specific biological pathways, showcasing the model’s ability to capture these diverse oncogenic mechanisms.

### Functional enrichment of novel candidate driver genes

To assess the ability of GRAFT to identify previously unknown cancer driver genes, an additional analysis was performed focusing on newly predicted genes not included in the known cancer driver gene set. After predicting probabilities for all genes using the trained model, the top 300 genes with the highest scores were selected. From this set, 122 known cancer driver genes were removed, resulting in a list of 178 novel candidate driver genes.

**Figure 7:**
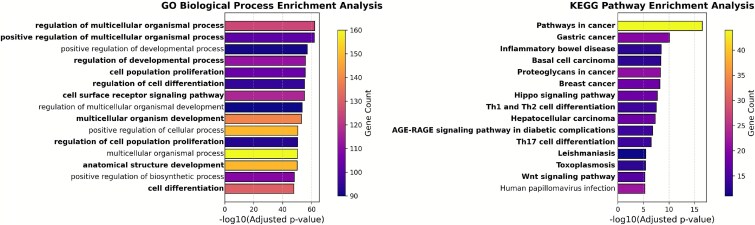
The enrichment results in two datasets, GO biological processes and KEGG pathways, for novel candidate driver genes identified by GRAFT. Top 15 enriched terms in each dataset, selected based on adjusted *P*-values, are displayed. Amon them, the cancer-related terms are emphasized in bold. The color intensity represents the number of associated genes in each term.

To investigate the biological relevance of these novel candidates, enrichment analysis was performed for GO biological processes and KEGG pathways using g:Profiler [38]. For each dataset, the top 15 terms were selected based on adjusted *P*-values and visualized as bar plots, where color intensity represented the number of associated genes. In Fig. 7, cancer-related terms were visually emphasized using bold font. These terms were identified through two complementary strategies. First, a set of cancer-related MeSH disease identifiers was obtained from the Comparative Toxicogenomics Database (CTD) [39] by collecting all descendant terms under the top-level cancer node in the MeSH hierarchy (MeSH:D009369) [40]. Terms from GO and KEGG were then mapped to these identifiers. Second, additional relevant terms were identified through keyword-based matching using cancer-specific terms such as cancer, tumor, and carcinoma. Terms matching either criterion were marked as cancer-associated and highlighted accordingly in the visualizations.

The enrichment analysis results provided substantial evidence supporting the biological relevance and potential clinical significance of the predicted genes. As shown in Fig. 7, several of the most enriched GO biological process terms are closely related to tumor development and progression. Terms such as regulation of multicellular organismal process, positive regulation of multicellular organismal process, and cell population proliferation are directly involved in tissue growth control, cell fate determination, and tumorigenesis. Deregulation of these processes has been widely implicated in cancer initiation and metastasis [4, 7, 41, 42]. Indeed, uncontrolled cell proliferation is a defining hallmark of cancer, and its rate often correlates with tumor grade, aggressiveness, and patient prognosis across various cancer types [43].

Most notably, the KEGG pathway enrichment analysis clearly identified several well-established cancer pathways, as shown in Fig. 7. Terms such as pathways in cancer, gastric cancer, basal cell carcinoma, proteoglycans in cancer, and breast cancer were significantly enriched among the predicted genes, indicating that the predictions are highly relevant to known cancer biology. These pathways are involved in key oncogenic events such as aberrant cell growth, apoptosis resistance, and extracellular matrix remodeling [44–46]. The identification of Hippo signaling pathway and Wnt signaling pathway is also particularly noteworthy, as both have been implicated in cancer stemness and organ size control and are frequently dysregulated in a variety of tumor types [47, 48]. Importantly, both the Wnt [49] and Hippo [50] signaling pathways are intensely studied as potential therapeutic targets in oncology. Aberrant signaling through these pathways is frequently linked to tumor initiation, progression, metastasis, drug resistance, and poor clinical outcomes, highlighting the potential clinical relevance of targeting components within these cascades [49, 50]. These findings collectively suggest that the predicted candidate genes are functionally enriched in core processes and pathways central to cancer pathogenesis. This provides further confidence in their biological relevance and potential roles as novel cancer drivers, potentially offering new avenues for diagnostic biomarker development and targeted therapeutic strategies in clinical oncology.

## Discussion and Conclusion

In this study, we introduced GRAFT, a novel deep learning framework that demonstrates a robust ability to predict cancer driver genes by synergistically integrating multi-view biological networks with multi-omics data. As shown in our comparative evaluations (Tables 2 and 3), GRAFT achieves highly competitive performance across both pan-cancer and numerous specific cancer types, generally matching or surpassing recent state-of-the-art methods. The effectiveness observed stems from the distinct architecture of GRAFT, designed to overcome limitations of previous approaches. Unlike methods relying on single networks or superficial fusion, GRAFT employs dedicated GCNs for multi-view feature extraction (PPI, GO, Pathway) coupled with an attention mechanism for adaptive fusion. Furthermore, it utilizes a Transformer backbone enhanced with a novel edge-attention bias, allowing it to capture global, long-range dependencies across the gene network while remaining explicitly sensitive to graph topology, thereby moving beyond the local constraints of conventional GNNs. This synergistic integration of multi-modal data and graph-aware global modeling likely enables GRAFT to capture subtle biological signals contributing to its strong performance.

Beyond predictive performance, GRAFT also provided insights into the biological characteristics of novel candidate genes, underscoring the power of a multifaceted approach to understanding complex diseases. A key finding of our study is the biological relevance of these novel candidates. Our functional enrichment analysis revealed that these genes, which are distinct from known drivers, are significantly enriched in critical cancer-related pathways. The strong enrichment in various oncogenic processes and signaling pathways suggests that the model effectively captures fundamental biological principles. This result validates the biological plausibility of our predictions, indicating that the model can discover novel genes that likely contribute to tumorigenesis through established mechanisms.

Furthermore, our analysis of the model’s attention mechanism provides insight into its decision-making process. Clustering of attention weights revealed that driver genes do not conform to a single pattern but rather exist as multiple archetypes, each characterized by a distinct reliance on specific network modalities. This indicates that GRAFT learns to identify diverse types of driver genes—from central hubs in interaction networks to key components of specific signaling pathways—showcasing a nuanced understanding of the varied roles genes play in cancer. Many previous approaches for driver gene prediction have relied on single data modalities or network types. GRAFT distinguishes itself by creating a more holistic representation, combining three distinct network views with multi-omics data. Moreover, our introduction of an edge attention bias specifically adapts the Transformer architecture for this context, allowing it to model global, long-range dependencies while remaining sensitive to the local graph topology. This represents a methodological advance beyond conventional GNN that primarily capture local neighborhood information.

Despite its promising results, this study has several limitations that open avenues for future research. The model’s performance is inherently dependent on the quality and completeness of the underlying biological databases, which are known to have ascertainment biases toward well-studied genes. Methodologically, while the GRAFT architecture is comprehensive, there are opportunities for further refinement. The current model relies on static biological networks, which may not fully represent the dynamic, context-specific nature of cellular interactions in different cancer subtypes; developing methods to integrate tissue-specific or dynamic graphs is a significant future challenge. Additionally, while our attention analysis offers a degree of interpretability, exploring more advanced techniques to elucidate the causal reasoning behind the Transformer’s predictions remains an important area for development. Future work could also explore integrating additional data modalities, such as epigenomics and proteomics, to enhance predictive accuracy and apply the framework to other complex diseases.

Ultimately, these future directions are motivated by a key translational goal: to bridge computational prediction with clinical application. The novel candidate genes identified by GRAFT serve as a strong foundation for the next phase of research, which will focus on their potential as therapeutic targets. We plan to conduct in-depth analyses of these genes to understand their roles in key oncogenic pathways and to develop models capable of predicting patient-specific responses to targeted therapies. By focusing on these aspects, we aim to contribute to the development of more effective precision therapies, ultimately improving outcomes for cancer patients.

## Supplementary Material

Supplementary_File_bbaf706

## Data Availability

The source codes and datasets are publicly accessible at https://github.com/spcho-dev/GRAFT.

## Notes

Key Points

- Graph-Aware Fusion Transformer (GRAFT) is a novel deep learning framework that predicts cancer driver genes by integrating multi-view biological networks and multi-omics data through a comprehensive architecture incorporating multi-view graph encoding, context-aware feature augmentation, and a transformer with an edge-attention bias.
- GRAFT employs an attention mechanism to dynamically fuse features from network-specific GCN encoders, allowing it to adaptively weight each biological view for individual genes.
- GRAFT achieves competitive performance with leading state-of-the-art methods in pan-cancer analysis, while consistently delivering superior predictive accuracy across numerous specific cancer types.
- Functional enrichment analysis validates the biological relevance of novel candidate driver genes, while the model’s learned representations effectively differentiate distinct archetypes of driver genes, providing new biological insights.

## References

[1] Dinstag G, Shamir R. Prodigy: personalized prioritization of driver genes. *Bioinformatics* 2020; 36:1831–9. 10.1093/bioinformatics/btz815 31681944 PMC7703777

[2] Anandakrishnan R, Varghese RT, Kinney NA et al. Estimating the number of genetic mutations (hits) required for carcinogenesis based on the distribution of somatic mutations. *PLoS Comput Biol* 2019; 15:e1006881. 10.1371/journal.pcbi.1006881 30845172 PMC6424461

[3] Shrestha R, Hodzic E, Sauerwald T et al. Hit’ndrive: patient-specific multidriver gene prioritization for precision oncology. *Genome Res* 2017; 27:1573–88. 10.1101/gr.221218.117 28768687 PMC5580716

[4] Vogelstein B, Papadopoulos N, Velculescu VE et al. Cancer genome landscapes. *Science* 2013; 339:1546–58. 10.1126/science.1235122 23539594 PMC3749880

[5] Martínez-Jiménez F, Muiños F, Sentís I et al. A compendium of mutational cancer driver genes. *Nat Rev Cancer* 2020; 20:555–72. 10.1038/s41568-020-0290-x 32778778

[6] Weinstein JN, Collisson EA, Mills GB et al. The cancer genome atlas pan-cancer analysis project. *Nat Genet* 2013; 45:1113–20. 10.1038/ng.2764 24071849 PMC3919969

[7] Hanahan D, Weinberg RA. Hallmarks of cancer: the next generation. *Cell* 2011; 144:646–74. 10.1016/j.cell.2011.02.013 21376230

[8] Dees ND, Zhang Q, Kandoth C et al. Music: identifying mutational significance in cancer genomes. *Genome Res* 2012; 22:1589–98. 10.1101/gr.134635.111 22759861 PMC3409272

[9] Lawrence MS, Stojanov P, Polak P et al. Mutational heterogeneity in cancer and the search for new cancer-associated genes. *Nature* 2013; 499:214–8. 10.1038/nature12213 23770567 PMC3919509

[10] Tamborero D, Gonzalez-Perez A, Lopez-Bigas N. Oncodriveclust: exploiting the positional clustering of somatic mutations to identify cancer genes. *Bioinformatics* 2013; 29:2238–44. 10.1093/bioinformatics/btt395 23884480

[11] Bashashati A, Haffari G, Ding J et al. Drivernet: uncovering the impact of somatic driver mutations on transcriptional networks in cancer. *Genome Biol* 2012; 13:1–14. 10.1186/gb-2012-13-12-r124 PMC4056374 23383675

[12] Lawrence MS, Stojanov P, Mermel CH et al. Discovery and saturation analysis of cancer genes across 21 tumour types. *Nature* 2014; 505:495–501. 10.1038/nature12912 24390350 PMC4048962

[13] Leiserson MDM, Vandin F, Hsin-Ta W et al. Pan-cancer network analysis identifies combinations of rare somatic mutations across pathways and protein complexes. *Nat Genet* 2015; 47:106–14. 10.1038/ng.3168 25501392 PMC4444046

[14] Cheng F, Zhao J, Zhao Z. Advances in computational approaches for prioritizing driver mutations and significantly mutated genes in cancer genomes. *Brief Bioinform* 2016; 17:642–56. 10.1093/bib/bbv068 26307061 PMC4945827

[15] Schulte-Sasse R, Budach S, Hnisz D et al. Integration of multiomics data with graph convolutional networks to identify new cancer genes and their associated molecular mechanisms. *Nat Mach Intell* 2021; 3:513–26. 10.1038/s42256-021-00325-y

[16] Peng W, Tang Q, Dai W et al. Improving cancer driver gene identification using multi-task learning on graph convolutional network. *Brief Bioinform* 2022; 23:bbab432. 10.1093/bib/bbab432 34643232

[17] Dazhi L, Zheng Y, Yi X et al. Identifying potential risk genes for clear cell renal cell carcinoma with deep reinforcement learning. *Nat Commun* 2025; 16:3591. 10.1038/s41467-025-58439-5 40234405 PMC12000451

[18] Deng C, Li H-D, Zhang L-S et al. Identifying new cancer genes based on the integration of annotated gene sets via hypergraph neural networks. *Bioinformatics* 2024; 40:i511–20. 10.1093/bioinformatics/btae257 38940121 PMC11211849

[19] Wang T, Zhuo L, Chen Y et al. ECD-CDGI: an efficient energy-constrained diffusion model for cancer driver gene identification. *PLoS Comput Biol* 2024; 20:e1012400. 10.1371/journal.pcbi.1012400 39213450 PMC11392234

[20] Zhao W, Xun G, Chen S et al. Modig: integrating multi-omics and multi-dimensional gene network for cancer driver gene identification based on graph attention network model. *Bioinformatics* 2022; 38:4901–7. 10.1093/bioinformatics/btac622 36094338

[21] Peng W, Zhou Z, Dai W et al. Multi-network graph contrastive learning for cancer driver gene identification. *IEEE Trans Netw Sci Eng* 2024; 11:3430–40. 10.1109/TNSE.2024.3373652

[22] Szklarczyk D, Nastou K, Koutrouli M et al. The string database in 2025: protein networks with directionality of regulation. *Nucleic Acids Res* 2025; 53:D730–7. 10.1093/nar/gkae1113 39558183 PMC11701646

[23] Kamburov A, Herwig R. Consensuspathdb 2022: molecular interactions update as a resource for network biology. *Nucleic Acids Res* 2022; 50:D587–95. 10.1093/nar/gkab1128 34850110 PMC8728246

[24] Gene Ontology Consortium . The gene ontology resource: 20 years and still going strong. *Nucleic Acids Res* 2019; 47:D330–8. 10.1093/nar/gky1055 30395331 PMC6323945

[25] Kanehisa M, Furumichi M, Tanabe M et al. KEGG: new perspectives on genomes, pathways, diseases and drugs. *Nucleic Acids Res* 2017; 45:D353–61. 10.1093/nar/gkw1092 27899662 PMC5210567

[26] Liberzon A, Birger C, Thorvaldsdóttir H et al. The molecular signatures database hallmark gene set collection. *Cell Syst* 2015; 1:417–25. 10.1016/j.cels.2015.12.004 26771021 PMC4707969

[27] Nishimura D . Biocarta. *Biotech Software & Internet Report: Comput Softw J Sci* 2001; 2:117–20. 10.1089/152791601750294344

[28] Fabregat A, Jupe S, Matthews L et al. The reactome pathway knowledgebase. *Nucleic Acids Res* 2018; 46:D649–55. 10.1093/nar/gkx1132 29145629 PMC5753187

[29] Köhler S, Gargano M, Matentzoglu N et al. The human phenotype ontology in 2021. *Nucleic Acids Res* 2021; 49:D1207–17. 10.1093/nar/gkaa1043 33264411 PMC7778952

[30] Repana D, Nulsen J, Dressler L et al. The network of cancer genes (NCG): a comprehensive catalogue of known and candidate cancer genes from cancer sequencing screens. *Genome Biol* 2019; 20:1–12. 10.1186/s13059-018-1612-0 30606230 PMC6317252

[31] Tate JG, Bamford S, Jubb HC et al. Cosmic: the catalogue of somatic mutations in cancer. *Nucleic Acids Res* 2019; 47:D941–7. 10.1093/nar/gky1015 30371878 PMC6323903

[32] Kim J, So S, Lee H-J et al. Digsee: disease gene search engine with evidence sentences (version cancer). *Nucleic Acids Res* 2013; 41:W510–7. 10.1093/nar/gkt531 23761452 PMC3692119

[33] Amberger JS, Bocchini CA, Scott AF et al. Omim. Org: leveraging knowledge across phenotype–gene relationships. *Nucleic Acids Res* 2019; 47:D1038–43. 10.1093/nar/gky1151 30445645 PMC6323937

[34] Chatzianastasis M, Vazirgiannis M, Zhang Z. Explainable multilayer graph neural network for cancer gene prediction. *Bioinformatics* 2023; 39:btad643. 10.1093/bioinformatics/btad643 PMC10636280 37862225

[35] Kong G, Wang J, Wang J. Gatomics: a novel multi-omics graph attention network model for cancer driver gene detection. In: ICASSP 2025-2025 IEEE International Conference on Acoustics, Speech and Signal Processing (ICASSP), Hyderabad, India, 2025, pp. 1–5. 10.1109/ICASSP49660.2025.10889716

[36] Deng C, Li H, Wang J. Improving cancer gene prediction by enhancing common information between the PPI network and gene functional association. *Proc AAAI Conf Artif Intell* 2025; 39:137–45. 10.1609/aaai.v39i1.31989

[37] Xiaorui S, Pengwei H, Li D et al. Interpretable identification of cancer genes across biological networks via transformer-powered graph representation learning. *Nat Biomed Eng* 2025; 9:371–89. 10.1038/s41551-024-01312-5 39789329

[38] Kolberg L, Raudvere U, Kuzmin I et al. G: Profiler—interoperable web service for functional enrichment analysis and gene identifier mapping (2023 update). *Nucleic Acids Res* 2023; 51:W207–12. 10.1093/nar/gkad347 37144459 PMC10320099

[39] Davis AP, Wiegers TC, Sciaky D et al. Comparative toxicogenomics database’s 20th anniversary: Update 2025. *Nucleic Acids Res* 2025; 53:D1328–34. 10.1093/nar/gkae883 39385618 PMC11701581

[40] Dhammi IK, Kumar S. Medical subject headings (mesh) terms. *Ind J Orthop* 2014; 48:443–4. 10.4103/0019-5413.139827 PMC4175855 25298548

[41] Malumbres M, Barbacid M. Cell cycle, CDKs and cancer: a changing paradigm. *Nat Rev Cancer* 2009; 9:153–66. 10.1038/nrc2602 19238148

[42] Bissell MJ, Hines WC. Why don’t we get more cancer? A proposed role of the microenvironment in restraining cancer progression. *Nat Med* 2011; 17:320–9. 10.1038/nm.2328 21383745 PMC3569482

[43] Matthews HK, Bertoli C, de Bruin RAM. Cell cycle control in cancer. *Nat Rev Mol Cell Biol* 2022; 23:74–88. 10.1038/s41580-021-00404-3 34508254

[44] Kanehisa M, Furumichi M, Sato Y et al. KEGG for taxonomy-based analysis of pathways and genomes. *Nucleic Acids Res* 2023; 51:D587–92. 10.1093/nar/gkac963 36300620 PMC9825424

[45] Hanahan D . Hallmarks of cancer: new dimensions. *Cancer Discov* 2022; 12:31–46. 10.1158/2159-8290.CD-21-1059 35022204

[46] Hynes RO . The extracellular matrix: not just pretty fibrils. *Science* 2009; 326:1216–9. 10.1126/science.1176009 19965464 PMC3536535

[47] Harvey KF, Zhang X, Thomas DM. The hippo pathway and human cancer. *Nat Rev Cancer* 2013; 13:246–57. 10.1038/nrc3458 23467301

[48] Clevers H, Nusse R. Wnt/$\beta $-catenin signaling and disease. *Cell* 2012; 149:1192–205. 10.1016/j.cell.2012.05.012 22682243

[49] Zhan T, Rindtorff N, Boutros M. Wnt signaling in cancer. *Oncogene* 2017; 36:1461–73. 10.1038/onc.2016.304 27617575 PMC5357762

[50] Ma S, Meng Z, Chen R et al. The hippo pathway: biology and pathophysiology. *Annu Rev Biochem* 2019; 88:577–604. 10.1146/annurev-biochem-013118-111829 30566373

