# Supplementary material for: GRAFT: a graph-aware fusion transformer for cancer driver gene prediction
Source: Brief Bioinform. 2026 Jan 11;27(1):bbaf706. doi: 10.1093/bib/bbaf706 (PMC12790624; doi:10.1093/bib/bbaf706)
Supplement: Supplementary_File_bbaf706 [file supplementary_file_bbaf706.pdf]

Supplementary materials for:

GRAFT: A graph-aware fusion transformer for cancer driver gene prediction

Sang-Pil Cho[1] and Young-Rae Cho[1,2,*]

[1]Division of Software, Yonsei University Mirae Campus, 1 Yeonsedae-gil, 26493, Gangwon-do, Republic of Korea

[2]Division of Digital Healthcare, Yonsei University Mirae Campus, 1 Yeonsedae-gil, 26493, Gangwon-do, Republic of Korea

*Corresponding author: youngcho@yonsei.ac.kr

Supplementary 1. Hyperparameter sensitivity analysis

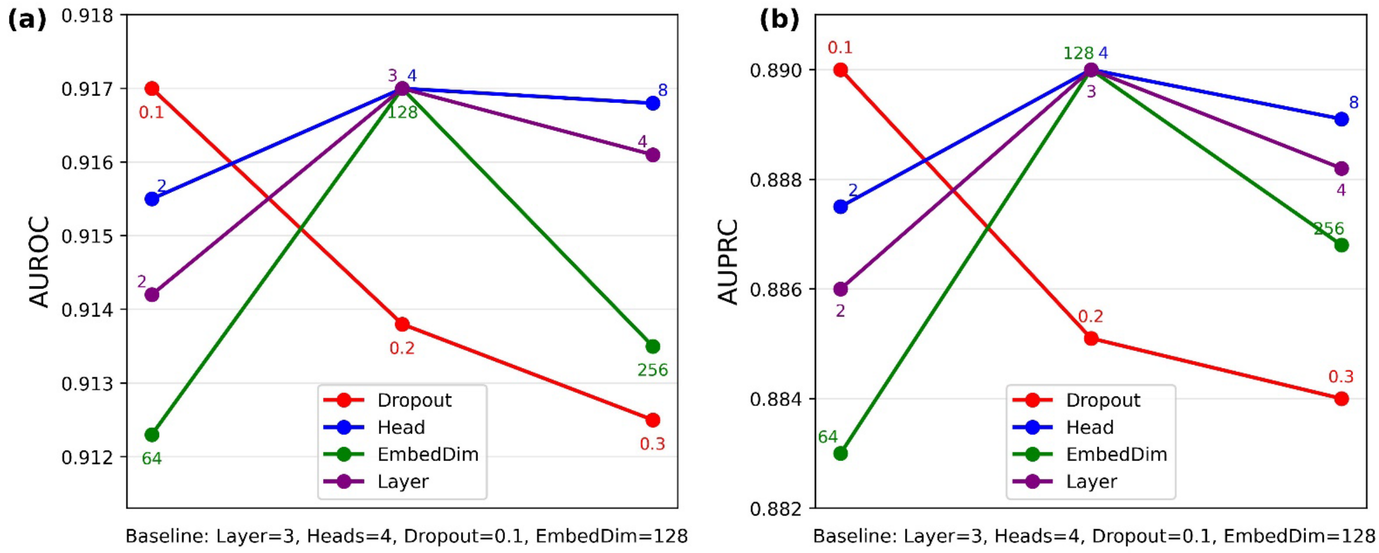

Figure S1. Hyperparameter sensitivity analysis for the pan-cancer model. Performance variation is shown by altering transformer layers, attention heads, embedding dimension, and dropout rate from the baseline configuration. Performance is evaluated by (a) AUROC and (b) AUPRC

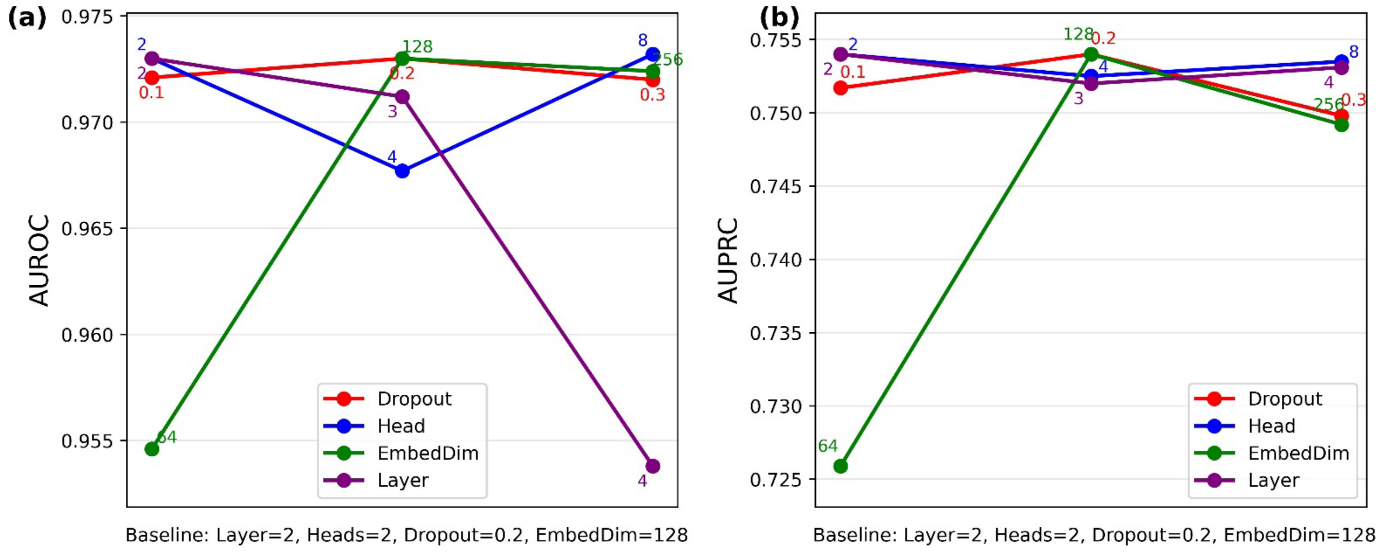

Figure S2. Hyperparameter sensitivity analysis for the cancer-specific model, using KIRC as a representative example. The analysis explores the impact of varying transformer layers, attention heads, embedding dimensions, and dropout rate on model performance. Performance is evaluated by (a) AUROC and (b) AUPRC.

To validate our model's hyperparameters, we conducted a sensitivity analysis for both the pan-cancer and a representative cancer-specific (KIRC) model, with results shown in Figure S1 and Figure S2, respectively.

For the pan-cancer model (Figure S1), the analysis confirmed the robustness of our baseline configuration (3 layers, 4 heads, 128 embedding dimension, 0.1 dropout), which achieved an AUROC of 0.9170 and AUPRC of 0.8900. The model demonstrated overall stability when varying layers, heads, and embedding dimensions, with the most significant performance decrease observed as the dropout rate was increased.

Similarly, for the KIRC-specific model (Figure S2), the baseline (2 layers, 2 heads, 128 embedding dimension, 0.2 dropout) proved to be a solid choice. While the model was also relatively stable, it showed distinct sensitivities. Increasing the model depth to 4 layers caused a noticeable drop in AUROC to 0.9538, suggesting a risk of overfitting. Furthermore, reducing the embedding dimension to 64 also led to a significant performance decline (AUROC 0.9546), indicating that a dimension of 128 is critical for capturing sufficient information in specific cancer datasets. These results collectively support our selected hyperparameters, demonstrating their effectiveness and stability for both pan-cancer and cancer-specific tasks.

Supplementary 2. Statistical significance analysis

Table S1. Statistical significance (P-values) of performance differences between GRAFT and baseline methods using the Wilcoxon signed-rank test on 10-fold cross-validation results.

Baseline Model	AUROC P-value (vs. GRAFT)	AUPRC P-value (vs. GRAFT)
$MODIG$	3.9062e-03	5.8594e-03
$EMGNN$	1.9531e-03	1.3487e-04
$GATOmics$	4.2969e-02	4.8828e-02
$ECD-CDGI$	1.6418e-03	2.5661e-03
$MNGCL$	2.1544e-02	7.8125e-03
$DISHyper$	8.4570e-01	1.9336e-01
$DISFusion$	6.7840e-01	2.3242e-01
$TREE$	1.1681e-03	1.3843e-04

Table S2. P-values from Wilcoxon signed-rank tests comparing the full GRAFT model against its ablation variants on 10-fold cross-validation results for the KIRC dataset.

Ablation Variant	AUROC P-value (vs. GRAFT)	AUPRC P-value (vs. GRAFT)
$GRAFT_{-PE}$	4.1016e-01	3.4180e-02
$GRAFT_{-CE}$	7.5531e-01	2.9531e-02
$GRAFT_{-GSA}$	2.9062e-03	1.1719e-04
$GRAFT_{-EAA}$	4.4414e-02	4.7428e-02
$GRAFT_{-NFE}$	1.9531e-03	3.9062e-03
$GRAFT_{-IF}$	4.8811e-02	2.4414e-02

As shown in Table S1, GRAFT's performance on the STRING dataset is statistically superior ($P < 0.05$) to six of the eight baseline methods, including MODIG, EMGNN, GATOmics, ECD-CDGI, MNGCL, and TREE. This holds true for both AUROC and AUPRC metrics in most cases, providing strong evidence for GRAFT's advanced predictive power. Notably, the performance difference between GRAFT and the other top-performing models, DISHyper and DISFusion, was not statistically significant ($P > 0.05$). This statistical evidence reinforces the conclusion presented in the main text: GRAFT is a state-of-the-art method, performing significantly better than the vast majority of existing approaches while being highly competitive with other leading models.

The results in Table S2 for the KIRC dataset underscore the importance of each architectural component in a data-limited setting. For the AUPRC metric, which is particularly informative for imbalanced datasets, the full GRAFT model demonstrated a statistically significant superiority over all six ablation variants ($P < 0.05$). The removal of the Gene Set Attention module (GRAFT$_{\scriptsize\text{–GSA}}$) led to the most significant performance drop, confirmed by the lowest P-value (1.17×10^{-4}).

For the AUROC metric, the full model was significantly superior to four of the six variants. The performance differences for GRAFT${\scriptsize\text{–PE}}$ and GRAFT${\scriptsize\text{–CE}}$ were not statistically significant, suggesting that while these positional and centrality encodings contribute to overall performance, other modules can partially compensate for their absence in terms of AUROC. This contrasts with the AUPRC metric, where the removal of these same two components did result in a statistically significant performance drop (Table S2). Overall, these tests provide statistical validation for our ablation study, confirming that the architectural design of GRAFT is robust and its key components are critical for optimal performance, especially in challenging, cancer-specific scenarios.

Supplementary 3. Quantitative evaluation of gene embedding clusters

Table S3. Results of quantitative metrics, including cluster quality scores and mean pairwise distances, for evaluating the learned gene embeddings.

Metric	Value	Interpretation
$Standard\ Clustering\ Quality\ Metrics$		
$Silhouette\ Score$	0.0786	$Higher\ is\ better\ (Range:\ -1\ to\ 1)$
$Davies-Bouldin\ Index$	2.2626	$Lower\ is\ better\ (Range: 0\ to\ \infty)$
$Calinski-Harabasz\ Index$	451.08	$Higher\ is\ better\ \ (Range: 0\ to\ \infty)$
$Mean\ Pairwise\ Cosine\ Distance$		
$Driver-Driver\ Distance$	0.6233	$Lower\ indicates\ higher\ cohesion$
$Nondriver-Nondriver\ Distance$	0.9754	
$Driver-Nondriver\ Distance$	1.0804	$Higher\ indicates\ better\ separation$

To quantitatively validate the visual separation of driver and non-driver genes observed in the UMAP visualization (Figure 2), we evaluated the structure of the high-dimensional gene embeddings. The evaluation was performed on the embeddings from each of the 10 cross-validation folds using several standard clustering quality metrics. The average results across all folds are summarized in Table S3, followed by a detailed explanation and analysis of each metric.

Analysis of Metrics

The evaluation metrics provide a multi-faceted view of the embedding space structure:

- Silhouette Score

The Silhouette Score measures how similar a data point is to its own cluster (cohesion) compared to other clusters (separation). For a single data point i, the score $s(i)$ is calculated as:

$$s(i) = \frac{b(i) - a(i)}{max\{a(i), b(i)\}}$$

where $a(i)$ is the mean distance from i to all other points in the same cluster, and $b(i)$ is the mean distance i to all points in the nearest neighboring cluster. The overall score is the average of $s(i)$ for all data points.

Analysis: Our model achieved a positive score of 0.0786. While modest, a positive score confirms that, on average, genes are closer to other genes of the same class than to genes of the opposing class. The low magnitude reflects the significant and expected overlap between the two groups, given the biological complexity and functional similarity between some driver and non-driver genes. It quantitatively supports the UMAP observation that the clusters are not perfectly discrete but possess a meaningful underlying structure.

- Davies-Bouldin Index (DBI)

The DBI validates clustering performance by combining a measure of within-cluster scatter with a measure of between-cluster separation. For a set of k clusters, the DBI is defined as:

$$DB = \frac{1}{k}\sum_{i=1}^{k} max_{j\neq i}\left(\frac{\sigma_i + \sigma_j}{d(c_i, c_j)}\right)$$

where σ_i is the average distance of all points in cluster i from the cluster centroid c_i, and $d(c_i, c_j)$ is the distance between centroids of clusters i and j A lower DBI value indicates better clustering.

Analysis: The score of 2.2626 suggests that the clusters are not perfectly compact or well-separated, which aligns with the conclusion from the Silhouette Score. It reflects that the within-cluster dispersion is not negligible compared to the between-cluster distance, consistent with the diffuse boundaries seen in the UMAP plot.

- Calinski-Harabasz Index (CHI)

Also known as the Variance Ratio Criterion, the CHI is the ratio of the between-cluster dispersion to the within-cluster dispersion. For k clusters, the score is defined as:

$$CH = \frac{SS_B/(k - 1)}{SS_W/(N - k)}$$

where SS_B is the overall between-cluster variance, SS_W is the overall within-cluster variance, and N is the total number of data points. A higher CHI score indicates denser, better-separated clusters.

Analysis: The relatively high score of 451.08 offers a complementary perspective. It indicates that even though the cluster boundaries overlap (as shown by low Silhouette and high DBI scores), the overall variance *between* the driver and non-driver clusters is substantially larger than the variance *within* each cluster. This suggests that the centers of mass for the two groups are well-separated in the embedding space.

- Mean Pairwise Cosine Distance

This metric provides a direct numerical summary complementing the distributions shown in the KDE plot (Figure 3). Cosine distance between two vectors u and v measures the angle between them, quantifying similarity independent of magnitude. It is calculated as:

$$Cosine\ Distance(u,v) = 1 - \frac{u \cdot v}{||u||\ ||v||} = 1 - \frac{\sum_{i=1}^{n} u_i v_i}{\sqrt{\sum_{i=1}^{n} u_i^2}\sqrt{\sum_{i=1}^{n} v_i^2}}$$

where a value closer to 0 indicates higher similarity (smaller angle), and a value closer to 2 indicates maximum dissimilarity. We computed the average cosine distance for all pairs within the driver group (Driver – Driver), within the non-driver group (Non-driver – Non-driver), and between the two groups (Driver – Non-driver).

Analysis: The results show a clear and ideal trend: $Distance(Driver - Driver) < Distance(Nondriver - Nondriver) < Distance(Driver - Nondriver)$. The substantially lower average distance for Driver–Driver pairs (0.6233) compared to Nondriver–Nondriver (0.9754) and Driver–Nondriver (1.0804) pairs quantitatively demonstrates the high cohesion and compactness of the learned driver gene cluster. This numerically proves that GRAFT successfully embeds driver genes much closer to each other than to any other genes, confirming that it has learned a semantically meaningful structure consistent with the KDE plot observations.
